# Prednisolone induces osteoporosis-like phenotypes via focal adhesion signaling pathway in zebrafish larvae

**DOI:** 10.1242/bio.029405

**Published:** 2018-07-15

**Authors:** Lei Huo, Lei Wang, Zhaoyao Yang, Pingyuan Li, Dechun Geng, Yaozeng Xu

**Affiliations:** 1 Department of Orthopedics, The First Affiliated Hospital of Soochow University, 188, Shi Zi Road, Suzhou 215006, China; 2 Department of Orthopedics, Suzhou Science & Technology Town Hospital, 1 Lijiang Road, New District, Suzhou 215010, China

**Keywords:** Zebrafish, GIOP, Focal adhesion signaling pathway, *itga10*, *itgbl1*

## Abstract

Patients taking glucocorticoid or glucocorticoid-like drugs for an extended period of time may develop osteoporosis, termed glucocorticoid-induced osteoporosis (GIOP). GIOP is the most common form of secondary osteoporosis, but the mechanism underlying its development is unclear. In the present study, we used prednisolone to treat zebrafish larvae to investigate GIOP. Our RNA deep-sequencing (RNA-seq) results show that prednisolone affects genes known to act in the extracellular region. Therefore the extracellular region, extracellular matrix, and collagen trimer might be involved in glucocorticoid-induced osteoporosis. Kyoto Encyclopedia of Genes and Genomes (KEGG) pathway analysis revealed that the focal adhesion signaling pathway is the most enriched signaling pathway in terms of differentially expressed genes (DEGs). In this pathway, *integrin subunit alpha 10* (*itga10*) and *integrin subunit beta like 1* (*itgbl1*), genes encoding two adapter proteins, were down-regulated in the prednisolone-treated larvae. Further experiments showed that prednisolone contributes to GIOP by down-regulating *itga10* and *itgbl1*.

## Abstract (precis)

**Summary:** Glucocorticoids may contribute to osteoporosis through effects on the extracellular region, the extracellular matrix and collagen trimer, and focal adhesion signaling pathway genes (*itga10* and *itgbl1*).

## INTRODUCTION

Glucocorticoids (GCs) are a class of corticosteroids that bind to glucocorticoid receptors (GRs), which are present in almost every vertebrate cell to regulate metabolism (Kadmiel and Cidlowski, 2013). GC-like drugs are very effective for treating inflammatory diseases (Straub and Cutolo, 2016). However, clinical data show that 50% of patients taking GCs or GC-like drugs for 6 months or longer develop osteoporosis, termed glucocorticoid-induced osteoporosis (GIOP), which is the most common form of secondary osteoporosis (Adinoff and Hollister, 1983; Reid, 1997; Seibel et al., 2013). Osteoporosis is found mainly in the elderly population resulting from an imbalance of osteoblast and osteoclast cells (Binkley and Krueger, 2005). Research has shown that GCs interfere with osteoblast differentiation and behavior both *in vitro* and *in vivo* (Weinstein et al., 1998). GCs have been found to increase apoptosis of mature osteoblasts and osteocytes and to impair differentiation of osteoblasts (Ohnaka et al., 2005) as well as bone formation (Conradie et al., 2007). Another study revealed that apoptosis of osteoblasts induced by GCs is related to the activation of glycogen synthase kinase 3β (GSK 3β), which plays a role in the Wnt signaling pathway (Yun et al., 2009). The Wnt signaling pathway plays an important role in controlling osteoblast differentiation and bone formation (Qiang et al., 2009). In addition to the Wnt signaling pathway, the bone morphogenetic protein (BMP) pathway was shown to be affected by GCs resulting in inhibition of osteoblast differentiation (Boden et al., 1997). It has been reported that patients treated with GCs exhibit a rapid increase in bone resorption followed by a chronic decrease in bone deposition. Osteoclast function is to break down bone tissue, and this is influenced by GCs (Elshal et al., 2013). In a mouse model, GC was shown to increase osteoclastogenesis after 7 days of treatment with the GC-like drug prednisolone, and decrease it by 60% after 21 days (Yao et al., 2008). Although several studies have focused on GIOP, the mechanism underlying its development is poorly understood.

The zebrafish is an excellent model in many areas of biological research, owing to their speed of development and fecundity (Lieschke and Currie, 2007). Zebrafish models have been used in bone research for many years, especially in the field of skeletal development, remodeling and growth (Witten et al., 2001). The GIOP zebrafish model was established in a 2006 study, where the authors exposed zebrafish larvae to different concentrations of prednisolone for 10 days post fertilization (dpf). Whole-mount skeletal staining was performed on fixed tissues. The authors demonstrated a statistically significant reduction in the area of stained bony tissue with 10 and 25 μM prednisolone exposure (Barrett et al., 2006). Additionally, adult zebrafish models have been applied in the study of GIOP. Adult zebrafish scales can easily be harvested with little stress to the zebrafish. A previous study used prednisolone to treat adult zebrafish and the scales were removed and allowed to regenerate. During their regeneration, scales were assessed and osteoblast and osteoclast activities monitored by expression profiling of cell-specific genes over time. The authors showed that prednisolone-treated zebrafish scales display enhanced osteoclast activity and matrix resorption, that prednisolone affects osteoblast and osteoclast gene expression and that zebrafish scales exhibit an osteoporosis-like imbalance in bone formation (de Vrieze et al., 2014). Another study used prednisolone-treated adult zebrafish to investigate the total mineral balance under prednisolone treatment using a time-dependent live dual staining method. The results showed that prednisolone-treated zebrafish display impaired resorption lacunae and a decrease in alkaline phosphatase (ALP) activity in scale scleroblasts. However, treatment with alendronate can rescue these phenotypes (Pasqualetti et al., 2015). Therefore, alendronate appears to effectively combat GIOP in fish as it does in humans.

RNA sequencing (RNA-seq), also called whole transcriptome shotgun sequencing (WTSS), uses next-generation sequencing (NGS) to detect and quantify RNA in a biological sample (Metzker, 2010). RNA-seq has been applied in many biomedical fields to investigate gene expression and expression differences with high throughput. Here, we established a zebrafish GIOP model and used RNA-seq to explore the mechanisms of GIOP. Our results show that treating zebrafish larvae with 25 μM prednisolone causes a significant delay in the mineralization process. This confirms that prednisolone-treated zebrafish larvae are a good model for studying GIOP. We then used RNA-seq to compare the prednisolone-treated group with the control group and found 1346 up-regulated genes and 1020 down-regulated genes, totaling 2366 differentially expressed genes (DEGs). Gene ontology (GO) enrichment analysis of the DEGs revealed the involvement of DEGs in the extracellular region, the extracellular matrix and the collagen trimer. Kyoto Encyclopedia of Genes and Genomes (KEGG) enrichment analysis of the DEGs revealed that the focal adhesion and ECM-receptor interaction signaling pathways were the most enriched. Additionally, we analyzed the signaling pathways of bone metabolism and found that the relative activity of the osteoblast signaling pathway was decreased, while the relative activity of the osteoclast pathway activity was increased. Next, we confirmed that the *integrin subunit alpha 10* (*itga10*) and *integrin subunit beta like 1* (*itgbl1*) genes, which belong to the focal adhesion and ECM-receptor interaction signaling pathways, contribute to GIOP. Our research provides new insights into GIOP.

## RESULTS

### Establishment of a zebrafish larva GIOP model induced by prednisolone

**Fig. 1.**
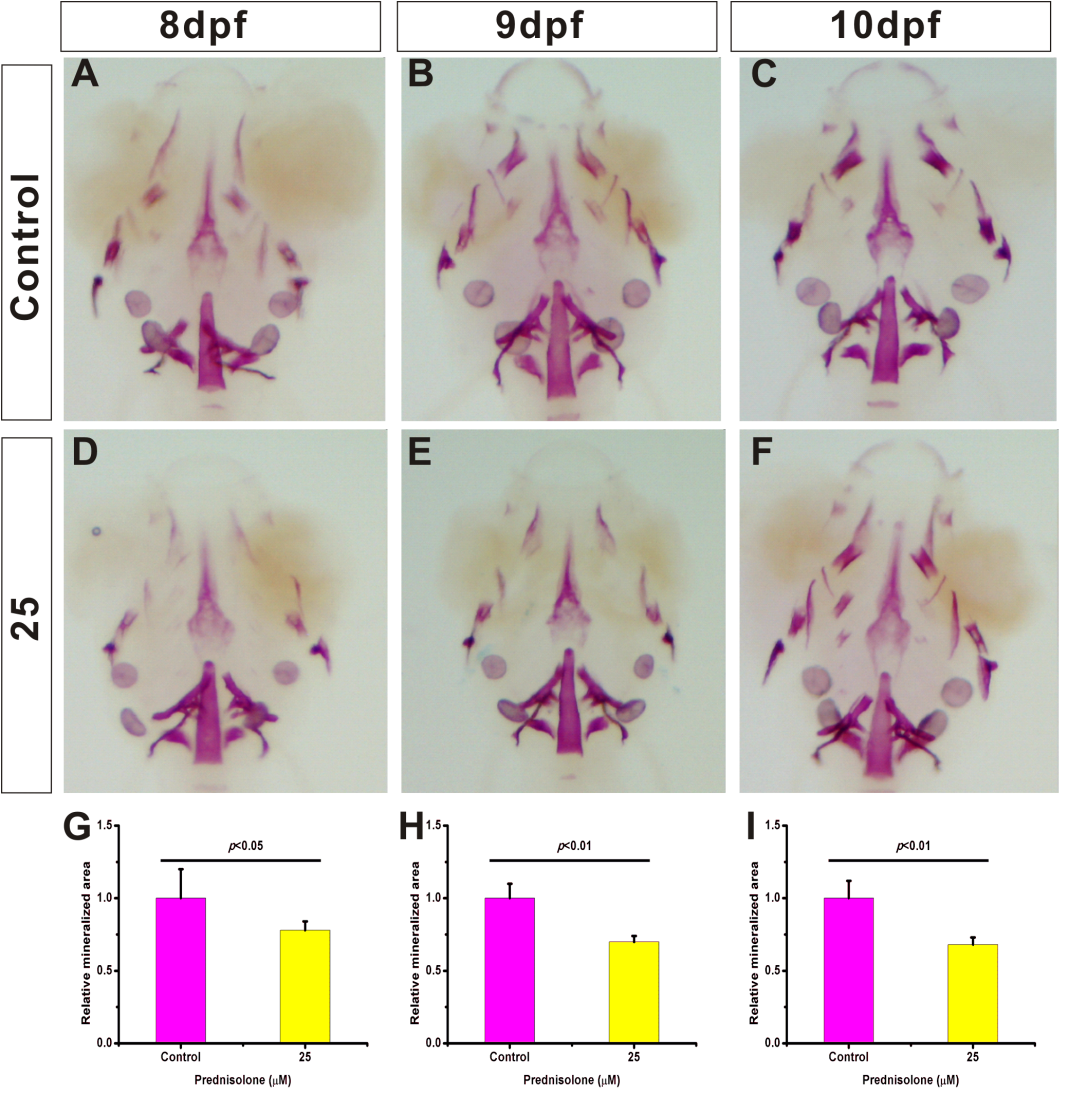
**Establishment of a zebrafish larva GIOP model using 25 μM prednisolone.** (A-F) 8−10 dpf zebrafish treated with 25 μM prednisolone. Whole mount skeletal staining was performed on fixed tissues. The mineralized tissue is stained purple and all other tissues are transparent. (G-I) Digital image analysis of stained area and staining density by assessment of fluorescence intensity. The stained mineralized tissue was quantified using analysis software. Mean values are plotted (*n*=10) and the Student's *t*-test was performed to determine statistical significance.

For establishing the zebrafish larva GIOP model, 5 dpf larva zebrafish were exposed to 25 µM prednisolone and incubated with the drug until collected. Next, samples were collected at 8 dpf, 9 dpf and 10 dpf. Whole-mount skeletal staining was performed on fixed tissue. The staining results showed that the area of stained bony tissue was decreased in the group exposed to 25 µM prednisolone compared with the Dimethyl Sulfoxide (DMSO) control groups (Fig. 1A-F). From 8 dpf, there was a significant decrease in staining in the exposure groups (Fig. 1G). However, over the course of the study, more severe differences were seen in the 9 dpf and 10 dpf larvae (Fig. 1H,I), indicating that the 25 µM prednisolone treatment can cause an osteoporosis-like phenotype in zebrafish larvae. These results are consistent with previous reports (Barrett et al., 2006). To investigate the mechanisms of this disorder, we harvested the 10 dpf 25 µM prednisolone-exposed group, and the DMSO control group for RNA-seq.

### RNA-seq data and differentially expressed genes

**Fig. 2.**
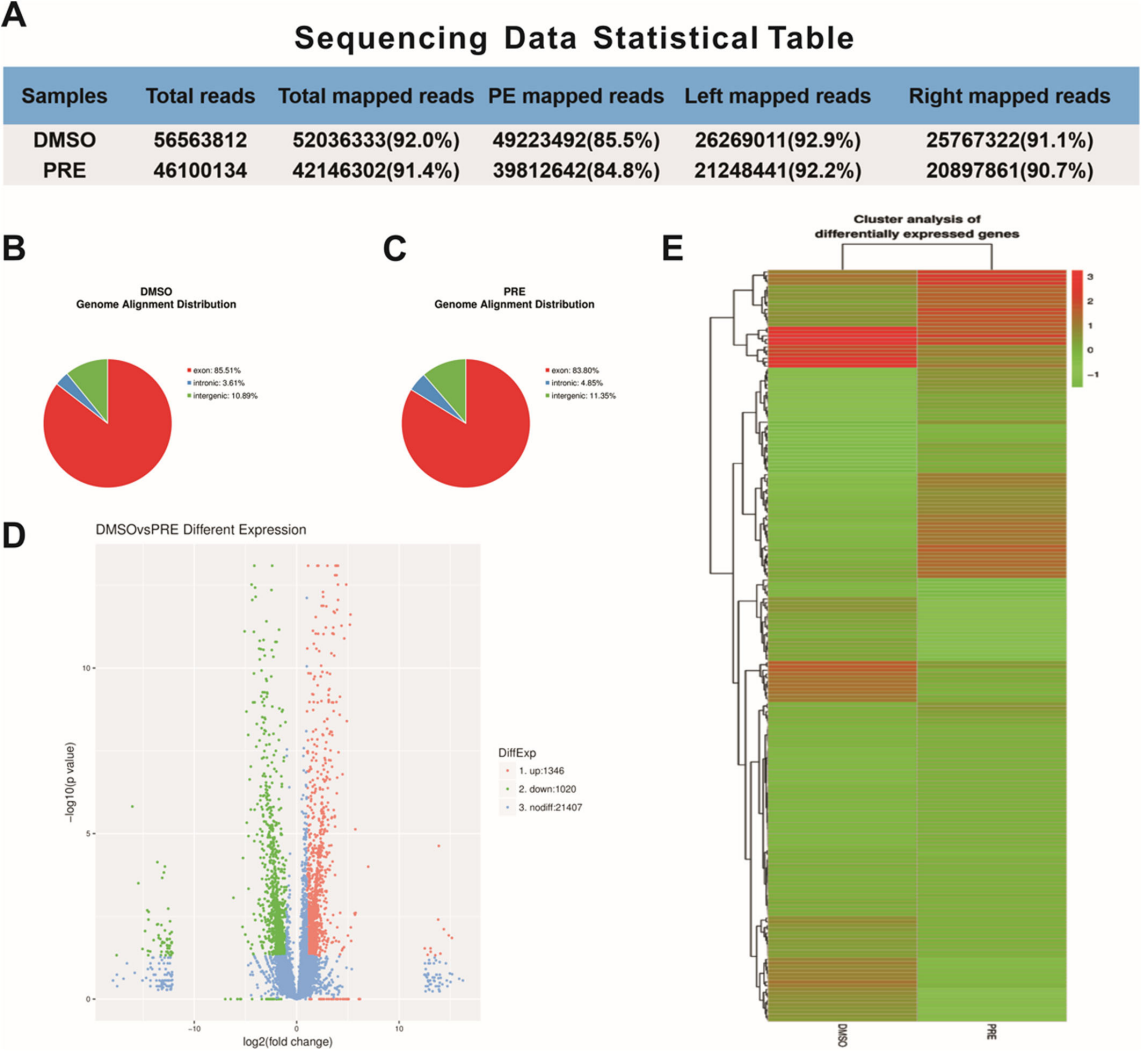
**Sequencing data and differential gene expression analysis.** (A) Comparison of sequencing data and reference sequences. (B,C) Based on the comparison of the sequencing data, and combined with the annotated documents of the reference sequence, the reads of the different positions of the reference sequence were compared, including the exon region (exon), intron region (intronic) and intergenic region (intergenic). (D) Volcano plot of differentially expressed genes. The criteria for screening differentially expressed genes were: |log2Ratio| ≥1 and q-value ≤0.05. (E) Heatmap of the top 200 differentially expressed genes.

RNA-seq is a useful approach for obtaining a complete set of transcripts from certain tissues at specific developmental stages or under certain physiological conditions (Nagarajan and Pop, 2010). In order to analyze the transcriptomes of the groups exposed to 25 µM prednisolone and to DMSO, two sequencing libraries were prepared and sequenced using the Illumina paired-end method. In total, 56,563,812 reads from the DMSO control group and 46,100,134 reads from the prednisone-treated group were obtained (Fig. 2A). The left map reads and right map reads were higher than the 90% cutoff. The genome alignment distribution in the DMSO-treated group was as follows: exonic region, 85.51%; intronic region, 3.61%; and intergenic region, 10.89% (Fig. 2B). In the prednisolone-treated groups, the genome alignment distribution was as follows: exonic region, 83.30%; intronic region, 4.85%; and intergenic region, 11.35% (Fig. 2C). These reads were then used for the *de novo* assembly and were sufficiently high for further analyses of throughput and sequencing quality.

To detect GIOP-related gene changes, we compared the expression levels of all genes between the control and the prednisolone-treated groups in our RNA-seq data based on gene expression profiles using |log2Ratio|≥1 and q-value ≤0.05 as the thresholds. The results show that there were several DEGs between the control and prednisolone-treated groups, with 1346 genes being up-regulated and 1020 genes being down-regulated (Fig. 2D). A volcano plot of differential gene expression shows all differentially expressed genes in Fig. 2D. All of the DEGs are listed in Table S3. The top 200 DEGs are plotted in a heatmap in Fig. 2E.

### Gene ontology enrichment of differentially expressed genes revealed that prednisolone affects bone metabolic processes, possibly via the extracellular matrix

**Fig. 3.**
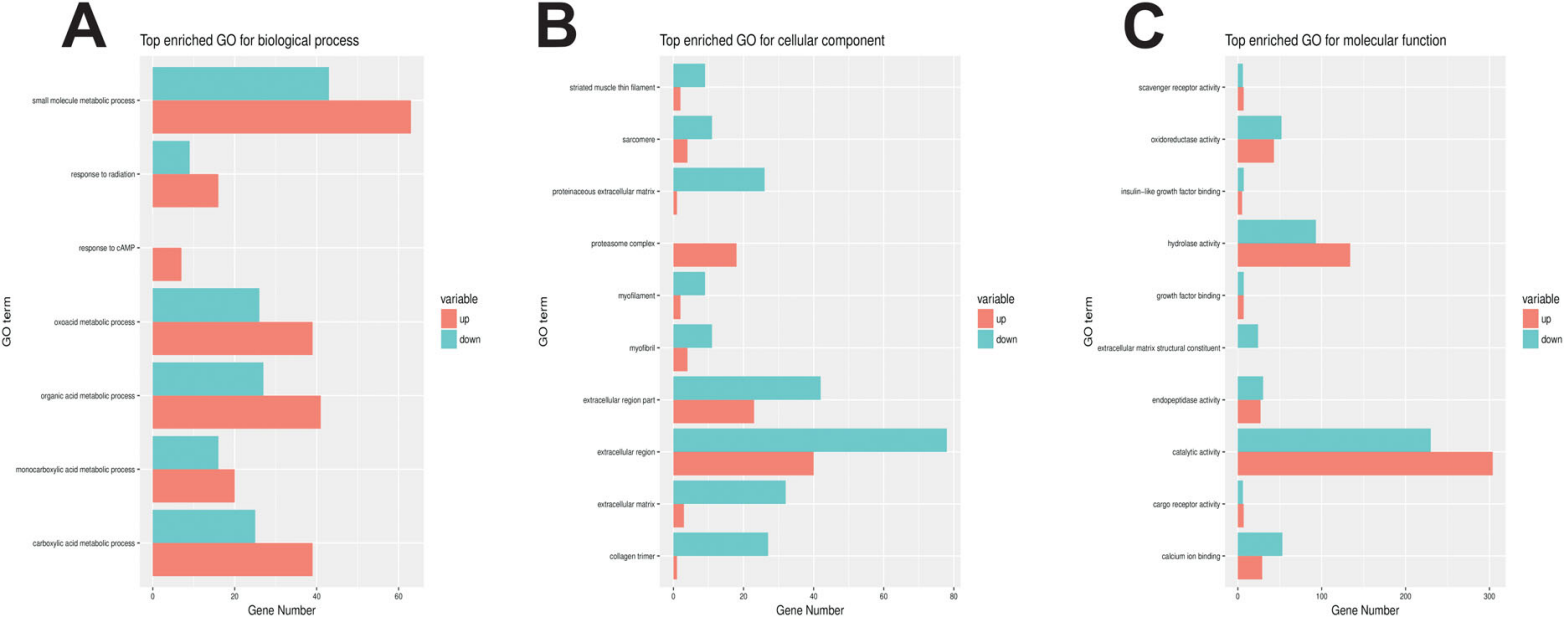
**Gene ontology for functional analysis of differentially expressed genes.** (A) GO functional analysis of differentially expressed genes according to biological process. (B) GO functional analysis of differentially expressed genes according to cellular component. (C) GO functional analysis of differentially expressed genes according to molecular function. The X-axis is the enriched GO term and the Y-axis is the number of significant differences in the term. Red indicates up-regulated genes and green indicates down-regulated genes.

Gene ontology (GO) is a major bioinformatics initiative to unify the representation of genes and gene product attributes across all species (Gene Ontology, 2008). It covers three domains: cellular components, molecular functions and the biological process (Diehl et al., 2007). In the biological process domain, the most highly enriched categories were the small molecule metabolic process, the response to radiation, and the monocarboxylic acid metabolic process (Fig. 3A). In the cellular component domain, the most highly enriched categories were extracellular region, extracellular matrix and collagen trimer (Fig. 3B). In the molecular function domain, the most highly enriched categories were catalytic activity, calcium ion binding, hydrolase activity and extracellular matrix structure constituency (Fig. 3C). All of the GO enrichments results are listed in Table S2. From the GO analysis, we found that prednisolone affects bone metabolic processes, possibly via the extracellular matrix.

### KEGG enrichment analysis of differentially expressed genes reveals that prednisolone affects the focal adhesion signaling pathway as well as the canonical osteoclast and osteoblast signaling pathways

**Fig. 4.**
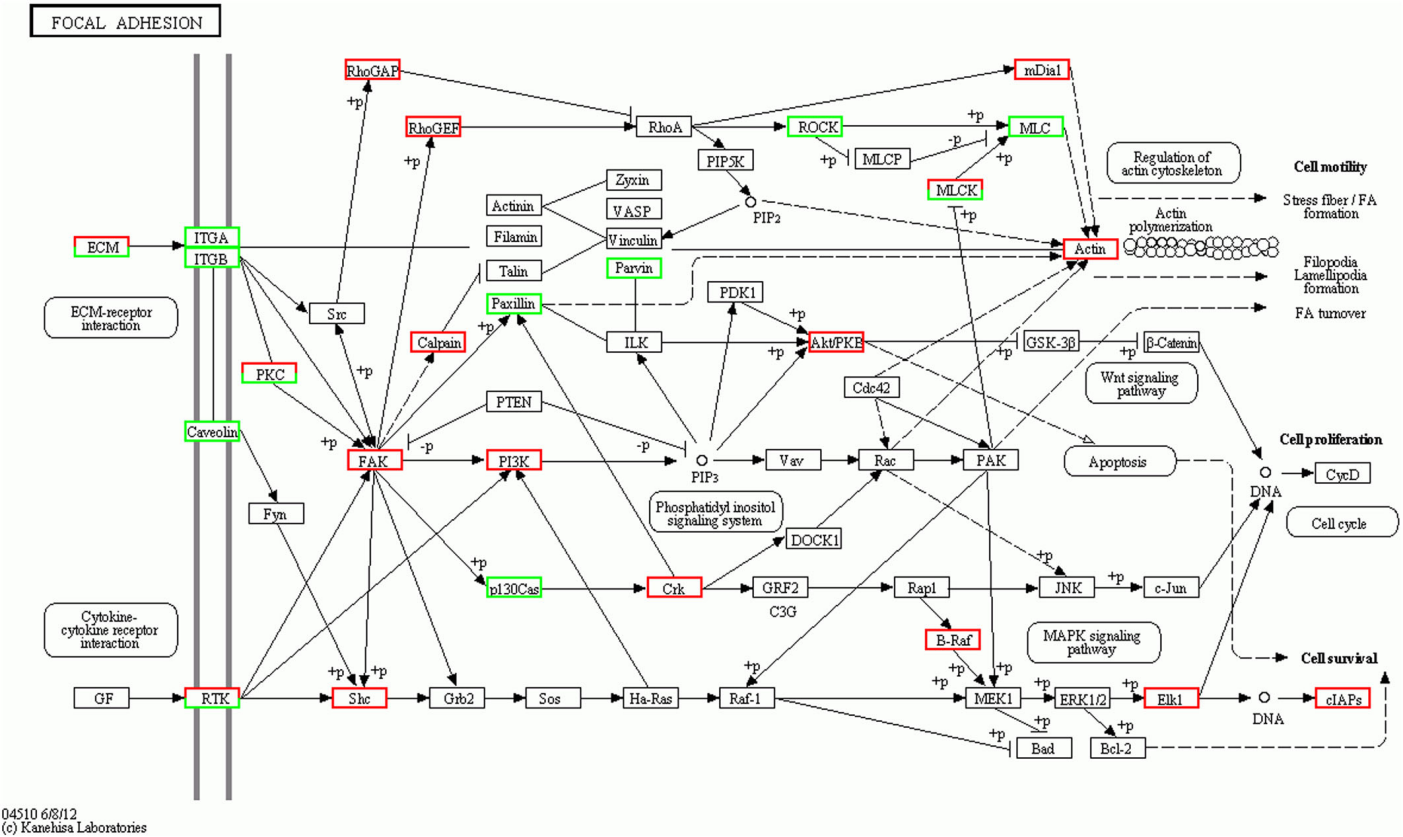
**KEGG pathway functional analysis of differentially expressed genes.** Focal adhesion signaling pathway was enriched by differentially expressed genes. Red indicates up-regulated genes and green indicates down-regulated genes.

KEGG is a database resource used to analyze the high-level functions and utility of biological systems from molecular-level information, and to further analyze the biological functions of genes (Kanehisa et al., 2008). The terms of the top KEGG pathways enriched in the DEGs were focal adhesion signaling pathway, ECM-receptor interaction signaling pathway, osteoclast difference signaling pathway and metabolic pathways (Fig. S1). All of the KEGG pathways enriched in the DEGs are listed in Table S5. In the focal adhesion and ECM-receptor interaction signaling pathways, there were 54 up-regulated genes and 124 down-regulated genes identified in the prednisolone-treated group compared with the control (Fig. 4). The *itga10* and *itgbl1* genes are important components of the focal adhesion signaling pathway (Robles and Gomez, 2006). The *itga10* gene encodes a protein which binds to collagen to transduce signals from the extracellular region to the intracellular region to regulate cell physiology (Camper et al., 1998). Its expression was found to be significantly decreased in the prednisolone-treated groups (Fig. 4). Collagen is the most important extracellular water insoluble fibrin, which is the backbone of the extracellular matrix (Yao et al., 2016). In our RNA-seq data, we found that almost all collagen-encoding genes were down-regulated (Table S4). These data demonstrate that prednisolone significantly affects focal adhesion and ECM-receptor interaction pathways.

Previously, the osteoclastogenesis signaling pathway was found to be significantly affected by prednisolone treatment in zebrafish larva (de Vrieze et al., 2014). Enriched signaling pathways in the DEGs included the osteoclast differentiation signaling pathway and the NF-kappa B signaling pathway, which are involved in osteoclast cell differentiation. In the NF-kappa B signaling pathway, 23 genes were up-regulated and three genes were down-regulated in the prednisolone-treated group compared with the DMSO-treated group. These include the *tnf*α, *il-1β* and *traf6* osteoclast genes, which were found to be significantly up-regulated (Fig. S2). In the osteoclast differentiation signaling pathway, 30 genes were up-regulated and eight genes were down-regulated in the prednisolone-treated group compared with the DMSO-treated group. Among these genes, the *Osteoprotegerin* (*opg*) gene, a key osteoclast differentiation factor and osteoporosis protein, was found (Fig. S3). These data reveal that prednisolone up-regulates members of the osteoclast signaling pathway.

In addition to modulating the osteoclastogenesis signaling pathway, the canonical osteoblastogenesis signaling pathway was found to be affected by prednisolone treatment in the zebrafish larva. Previous studies revealed that prednisolone causes a decrease in the number of osteoblasts (Sato et al., 2015). Therefore, we examined whether the osteoblastogenesis signaling pathway was present in the KEGG enrichment analysis of the DEGs. In the Wnt signaling pathway, there were 13 up-regulated genes and 10 down-regulated genes in the prednisolone-treated group compared with the DMSO-treated group. Among these genes, an important regulator, *wnt5b*, was significantly down-regulated (Fig. S4 and Table S4). In the MAPK signaling pathway, there were 17 up-regulated genes and 51 down-regulated genes in the prednisolone-treated group compared with the DMSO-treated group (Fig. S5 and Table S3). Among these genes were *fgf6a*, *fgf11b*, *fgfbp2b* and *fgfrl1b*, which have been reported to be key regulators of bone remolding (Ornitz and Marie, 2002; Naganawa et al., 2008). These data indicate that prednisolone down-regulates the osteoblast signaling pathway.

### The down-regulation of *itga10* and *itgbl1* contribute to cause the osteoporosis-like phenotype

**Fig. 5.**
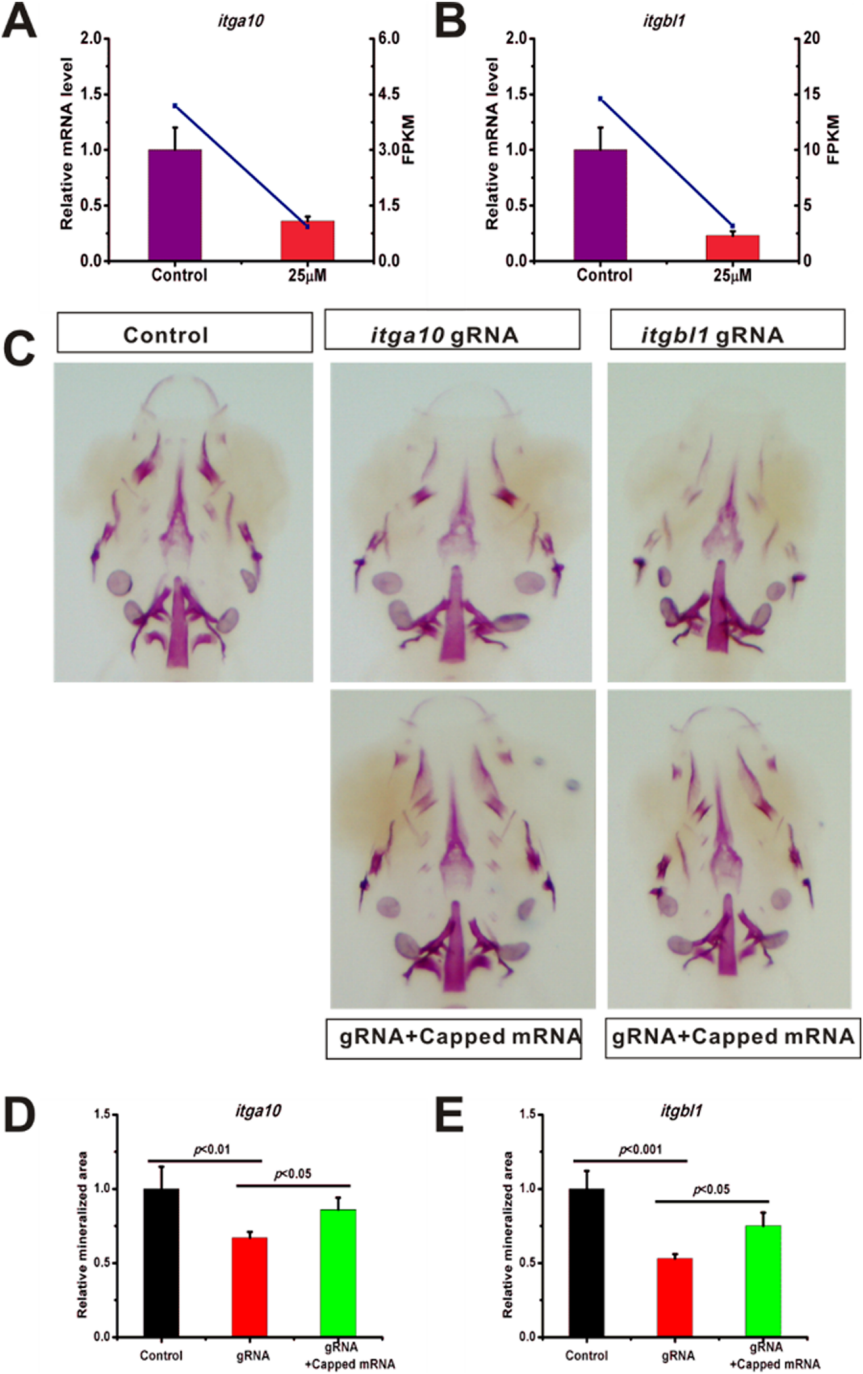
**Osteoporosis-like phenotypes caused by prednisolone through *itga10* and *itgbl1*.** (A) qRT-PCR confirmed that *itga10* expression is down-regulated by prednisolone. (B) qRT-PCR confirmed that itgbl1 expression is down-regulated by prednisolone. (C) The gRNAs for *itga10* and *itgbl1* were designed and injected into single-cell embryos. Whole mount skeletal staining was performed on fixed tissues. (D,E) Digital image analysis of stained area, and staining density by fluorescence intensity measurement. The stained mineralized tissue was quantified using analysis software. Mean values are plotted (*n*=5) and the Student’s *t*-test was performed to determine statistical significance.

Through the GO and KEGG analyses, we identified that the extracellular matrix (extracellular region, extracellular matrix and collagen trimer) and extracellular matrix-related signaling pathways (focal adhesion and ECM-receptor interaction signaling pathways) were the most enriched in the DEGs. We therefore investigated whether the extracellular matrix is involved in GIOP. The *itga10* and *itgbl1* genes are key regulators in the focal adhesion and ECM-extracellular interaction pathways, and were identified through RNA-seq to be down-regulated. Next, we investigated whether these two genes participate in the bone metabolism process. First, we performed qRT-PCR to validate the RNA-seq results indicative of down-regulation in the expression of *itga10* and *itgbl1* with 25 µM prednisolone treatment compared with DMSO treatment (Fig. 5A,B). The results show that the expression of *itga10* and *itgbl1* was significantly decreased in the prednisolone-treated group compared with the DMSO-treated group.

CRISPR/CRISPR-associated nuclease 9 (Cas9) is a recently developed revolutionary gene editing technology which is able to effectively manipulate almost every gene (Chang et al., 2013; Mali et al., 2013). Therefore, we used CRISPR/Cas9 to disrupt *itga10* and *itgbl1* expression *in vivo* to explore their functions in bone metabolism. The gRNAs for *itga10* and *itgbl1* were designed for exon 8 and exon 2 respectively. The Cas9 capped mRNA and gRNA were injected into single-cell stage fertilized eggs and saline was injected into eggs in the control group. The cutting efficiency was assessed and found to be 83% and 86% for *itga10* and *itgbl1*, respectively (Fig. S6). When the injected fish reached 10 dpf, whole mount skeletal staining was performed. We found that the skeleton was significantly reduced in both *itga10* and *itgbl1* mRNA injected groups compared with control groups (Fig. 5C-E). To further examine the roles of *itga10* and *itgbl1*, the exogenously capped mRNAs of both *itga10* and *itgbl1* were co-injected with the gRNA and Cas9 capped mRNA. The capped mRNA is easily degradable in the larva fish. To check whether the capped mRNA was present in the 10 dpf larvae, *itga10* and *itgbl1* mRNA fused to mCherry was injected into eggs. We found a weak but obvious mCherry fluorescence in the 10 dpf larvae (Fig. S7). Next, we performed whole-mount skeletal staining experiments. The results show that *itga10* and *itgbl1* mRNA partially rescued the reduced skeletal phenotype (Fig. 5C-E). These data reveal that the down-regulation of *itga10* and *itgbl1* contributes to causing the osteoporosis-like phenotype.

### Exogenous *itga10* and *itgbl1* capped mRNAs partially rescue the prednisolone-induced osteoporosis-like phenotype

**Fig. 6.**
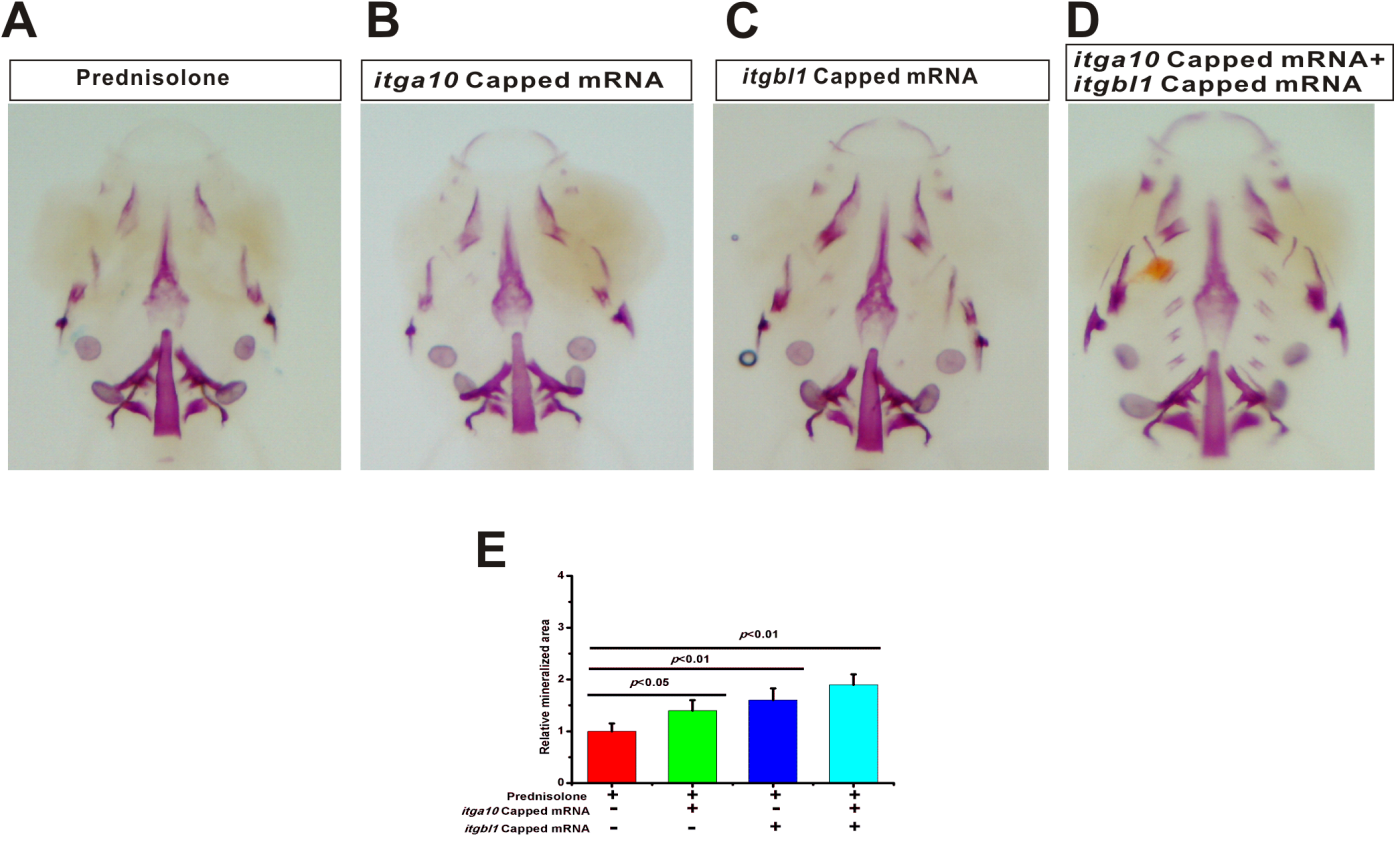
**Exogenous *itga10* and *itgbl1* capped mRNA rescue the osteoporosis-like phenotype of prednisolone.** (A) After treatment with 25 µM prednisolone, whole mount skeletal staining was performed at 10 dpf. (B) *itga10* capped mRNA was injected into single-cell embryos, larvae were treated with 25 μΜ prednisolone at 6 dpf and whole mount skeletal staining was conducted at 10 dpf. (C) *itgbl1* capped mRNA was injected into single-cell embryos. Larvae were treated with 25 μΜ prednisolone at 6 dpf, and whole mount skeletal staining was conducted at 10 dpf. (D) *itga10* and *itgbl1* capped mRNAs were co-injected into single-cell embryos, larvae were treated with 25 μΜ prednisolone at 6 dpf, and whole mount skeletal staining was conducted at 10 dpf. (E) Digital image analysis of stained area and staining density by fluorescence intensity measurement, and the stained mineralized tissue was quantified using analysis software. Mean values are plotted (*n*=5) and the Student's *t*-test was performed to determine statistical significance.

To determine whether exogenous *itga10* and *itgbl1* mRNA could rescue the osteoporosis-like phenotype caused by prednisolone, *itga10* and *itgbl1* capped mRNA was injected into single-cell fertilized eggs. Larvae were exposed to 25 µM prednisolone at 5 dpf. At 10 dpf, larvae were collected and whole mount skeletal staining was performed. The results show that *itga10* and *itgbl1* capped mRNA, both individually and double injected (Fig. 6A-C), increased the skeletal staining compared with the saline-injected control larvae (Fig. 6D). These results reveal that increasing *itga10* and *itgbl1* mRNA may partially rescue the prednisolone-induced osteoporosis-like phenotype.

## DISCUSSION

GCs signal through the GR, thereby affecting the expression of many genes (Reichardt et al., 1998). The GR gene, *gr*, is expressed in all tissues, including bone (Abu et al., 2000). GR signaling involves genomic and non-genomic pathways, and the pathophysiology of GIOP is highly complex (Revollo and Cidlowski, 2009). The zebrafish is an excellent model for studying GIOP. Although much research has been conducted in this field, the mechanisms of GIOP are still unclear. Here, we used 10 dpf zebrafish larvae to explore the causes of GIOP. Three major findings are reported in the present study. (1) The extracellular matrix may contribute to GIOP. (2) The focal adhesion signaling pathway may be involved in GIOP via the *itga10* and *itgbl1* integral transmembrane glycoproteins. (3) Prednisolone significantly increased the activities of the osteoclast and NF-kappa B signaling pathways.

The zebrafish model has been employed in the study of GIOP for many years. In 2006, Barrett and coworkers created the larva zebrafish model of GIOP, and reported that prednisolone causes a delay in the mineralization of the internal skeleton of zebrafish larvae (Barrett et al., 2006). Scales of adult zebrafish are a useful tissue for research of GIOP (de Vrieze et al., 2014). The extracellular matrix may be involved in the development of GIOP. To better understand the mechanisms of GIOP, we employed RNA-deep sequencing technology to investigate how prednisolone causes osteoporosis. GO enrichment analysis showed that the extracellular region, extracellular matrix and collagen trimer are the top enrichment categories in the cellular components domain. The extracellular matrix, which is synthesized by animal cells, and secreted into the extracellular space, is distributed on the surface of cells or between cells (Meredith et al., 1993). It contains polysaccharides, proteins and proteoglycans. These materials form a complex network structure, support and connect tissue structures, regulate the position of tissues, and are involved in numerous cell physiological activities (Geiger et al., 2001). In the bone, collagen composes approximately 90% of the organic matrix, and the basic characteristics of bone stability and plasticity are attributed to collagen, which gives the bone a high tensile strength (Ferreira et al., 2012). Collagen also provides mineral crystallization, which adds to the high tensile strength (Viguet-Carrin et al., 2006). Study of collagen matrix is therefore important to reveal the pathogenesis of osteoporosis (Sroga and Vashishth, 2012). Our deep sequencing results revealed that expression of almost all of the collagen genes are down-regulated in our GIOP model (Table S2). However, the mechanism by which prednisolone affects the expression of collagen genes, or the extent to which this contributes to osteoporosis, is unknown. The extracellular matrix and collagen trimer determine the extracellular matrix. The regulation of bone physiology by its extracellular matrix is crucial for the normal regulation of structure and function. And changes in the microenvironment could affect bone tissue and lead to osteoporosis, though this requires further study.

The focal adhesion signaling pathway is a key signaling pathway in osteoporosis caused by prednisolone. Through KEGG pathway enrichment analysis, the focal adhesion signaling pathway was found to be the most enriched in the DEGs. Focal adhesions mediate the interactions between the cell and the extracellular matrix. Focal adhesion is the process by which cells anchor themselves via the extracellular matrix (Burridge et al., 1988). This dynamic anchor causes cell adhesion via integrin-binding in the extracellular matrix. The cytoplasmic end of integrin proteins connects with actin filaments through adapter proteins. Focal adhesion also plays a role in the signal transduction process. However, how the alteration of the extracellular matrix influences intracellular processes via focal adhesion is still unclear. Two important genes in the focal adhesion signaling pathway were identified in our deep sequencing results: *itga10* and *itgbl1*. These two genes encode the integrin alpha and beta chains. Integrins are integral transmembrane glycoproteins composed of non-covalently linked alpha and beta chains (Hughes, 1992). They participate in cell adhesion as well as in cell-surface mediated signaling. To investigate the role of *itga10* and *itgbl1*, Cas9 was employed to disrupt these genes. When the *itga10* and *itgbl1* genes were disrupted, we found a similar phenotype as with prednisone-treated zebrafish larvae. To investigate the role of *itga10* and *itgbl1* in GIOP, we rescued the GIOP phenotype by injection of *itga10* and *itgbl1* capped mRNA. We found that *itga10* and *itgbl1* mRNA rescued the GIOP phenotype. Therefore, our results demonstrate that *itga10* and *itgbl1* play a role in GIOP.

The activity of the osteoclast signaling pathway significantly increased after prednisolone treatment. Previous studies revealed that prednisolone causes GIOP by decreasing the activity of osteoblasts in a mouse model (Giuliani et al., 1998). Recently, one study reported that prednisolone enhances osteoclast activity and matrix resorption (de Vrieze et al., 2014). These findings are consistent with our results. Another report showed that alendronate rescued the osteoporotic phenotype in a model of GC-induced osteoporosis in adult zebrafish scales (Pasqualetti et al., 2015). In our deep sequencing results, KEGG enrichment analysis of the DEGs shows that the activities of the osteoclast signaling pathway and NF-kappa B signaling increased. Almost all of the DEGs in the NF-kappa B signaling pathways exhibited increased expression. In zebrafish, enzymatic detection of tartrate-resistant alkaline phosphatase (TRAP) reveals the presence of osteoclasts from 20 dpf, but not earlier, suggesting that these cells are not present at the larval stages (Witten et al., 2001). Based on our deep sequencing results, it can be suggested that osteoclasts are present at 10 dpf, and possibly earlier. At this time point, prednisolone treatment increased osteoclast activity, which may cause osteoporosis-like phenotype.

## CONCLUSION

We conclude that prednisolone causes an osteoporosis-like phenotype via the focal adhesion signaling pathway and the alteration of extracellular matrix signaling in the cell by the *itga10* and *itgbl1* integral transmembrane glycoproteins. Prednisolone significantly increased the activity of osteoclast signaling and the NF-kappa B signaling pathway. Therefore, the results of our study provide further insights into GIOP.

## MATERIALS AND METHODS

### Fish maintenance and prednisone treatment procedure

All procedures were approved by the Soochow University Animal Care and Use Committee and were in accordance with governmental regulations of China. Adult zebrafish were raised in a re-circulation water system under 14/10 h light/dark (L/D) cycles at 28°C and fed three times per day. To produce embryos, male and female zebrafish were paired in the evening and spawning occurred the next day within 1 h after the lights were turned on. Embryos were placed in 10 cm Petri dishes with egg water containing Methylene Blue (0.3 ppm) and raised in a light-controlled (14/10 h L/D) incubator at 28°C. After 5 dpf, larvae were treated with 25 µM prednisone, while sibling larvae were treated with DMSO (0.1%, v/v) and used as the control group. For each treatment group, zebrafish larvae were raised in Petri dishes, and treatment solutions were changed daily until the time of sampling. At 8 dpf, 9 dpf and 10 dpf, samples were collected to perform whole-mount skeletal staining.

### Whole-mount skeletal staining

Zebrafish larvae were collected and fixed in 2% paraformaldehyde (PFA) overnight and trypsinized with 1% trypsin in a 35% saturated sodium borate solution overnight to remove the skin and muscles. After washing with 10% glycerol/0.5% KOH, larvae were stained with 0.02% Alizarin Red stain/10% glycerol/0.5% KOH overnight, and then washed twice with 50% glycerol/0.1% KOH overnight. Images were acquired with a stereomicroscope (M165FC; Leica, Frankfurt, Germany). Digital image analysis was performed to quantify stained area and staining density.

### RNA isolation, cDNA synthesis and quantitative Real-Time PCR

Total RNA was extracted from a pool of 25 larvae using Trizol (Invitrogen) reagent and reverse transcribed into cDNA with Superscript III Reverse Transcriptase (Invitrogen). Quantitative Real-Time PCR (qRT-PCR) was performed using an ABI Step-One Plus instrument with the SYBR (TaKaRa, Dalian, China) system with 40 cycles of 95°C for 10 s, and 58°C for 30 s. Each qRT-PCR assay was conducted with three independent biological samples and each sample was performed in triplicate. Three housekeeping genes (*β-actin*, *ef1α* and *gapdh*) were selected. Results show that *β-actin* was the most suitable reference gene (Table S1). All expression levels were normalized to the expression level of the housekeeping gene *β-actin*. Relative mRNA expression levels are expressed as 2^ΔΔCt^. The calculations were carried out with Microsoft Office Excel. Primer sequences are listed in the Supplementary Information.

### Deep sequencing analysis

RNA quantification was assessed using a Nano Drop ND-1000 UV/Vis-Spectrophotometer (Nano Drop Technologies, Boston, USA) and qualification of RNA degradation and contamination was monitored on 1% agarose gels. RNA purity was assessed using the NanoPhotome spectrophotometer (IMPLEN, West Lake Village, CA, USA). RNA concentration was measured using the Qubit^®^ RNA Assay Kit in a Qubit^®^2.0 Fluorimeter (Life Technologies). RNA integrity was assessed using the RNA Nano 6000 Assay Kit with the Agilent Bioanalyzer 2100 system (Agilent Technologies, Santa Clara, CA, USA).

### Library preparation for transcriptome sequencing

A total of 3 μg RNA per sample was used as the input material for the RNA sample preparations. Two sequencing libraries (control and treated) were generated using NEBNextUltra RNA Library Prep Kit for Illumina [New England Biolabs (NEB), Ipswich, MA, USA], which were added to attribute sequences to each sample. Briefly, mRNA was purified from total RNA using poly-T oligo-attached magnetic beads. Fragmentation was carried out using divalent cations under elevated temperature in NEBNext First Strand Synthesis Reaction Buffer (5×) (Invitrogen). First strand cDNA was synthesized using random hexamer primers and M-MuLV Reverse Transcriptase (RNase H−). Second strand cDNA synthesis was subsequently performed using DNA Polymerase I and RNase H. The remaining overhangs were converted into blunt ends via exonuclease/polymerase activities. After adenylating the 3′ ends of DNA fragments, NEBNext Adaptor with a hairpin loop structure was ligated to prepare for hybridization. In order to preferentially select cDNA fragments of ∼150−200 bp in length, the library fragments were purified with AMPure XP system (Beckman Coulter, Brea, CA, USA). Subsequently, 3 µl of USER Enzyme (NEB) was used with size-selected, adaptor-ligated cDNA at 37°C for 15 min followed by 5 min at 95°C before performing the PCR reaction. Then, PCR was performed with Phusion High-Fidelity DNA polymerase, Universal PCR primers and Index (X) Primer. Finally, PCR products were purified (AMPure XP system; Beckman Coulter) and library quality was assessed with the Agilent Bioanalyzer 2100 system.

### Clustering and sequencing

The clustering of the index-coded samples was performed on a cBot Cluster Generation System using TruSeq PE Cluster Kit v3-cBot-HS (Illumina, San Diego, CA, USA) according to the manufacturer's instructions. After cluster generation, the library preparations were sequenced on a Hiseq 2000 platform (Illumina) and paired-end reads were generated.

### Differential expression analysis

Prior to differential gene expression analysis, the read counts for each sequenced library were adjusted with the edgeR program package (Biomarker, Beijing, China) through one scaling normalized factor. Differential expression analysis between samples was performed using the DEGseq R package (Biomarker). *P*-values were adjusted using q value. *P* value <0.005 and |log 2 (fold change)| >1 were set as the thresholds for significant differential expression.

### Production of gRNA and Cas9 mRNA

gRNAs were transcribed using the *DraI*-digested gRNA expression vectors as templates and the MAXIscript T7 kit (Life Technologies). The Cas9 mRNA was transcribed using a *PmeI*-digested Cas9 expression vector and the mMESSAGE mMACHINE T7 ULTRA kit (Life Technologies). Following completion of transcription, the poly (A) tailing reaction and DNase I treatment were performed according to the manufacturer's instructions. Both the gRNA and the Cas9-encoding mRNA were then purified by LiCl precipitation and re-dissolved in RNase-free water.

### Microinjection of zebrafish embryos and evaluation of nuclease-associated toxicity

Guide RNA (gRNA) and Cas9-encoding mRNA were co-injected into one-cell stage zebrafish embryos. Unless otherwise indicated, each embryo was injected with 2 nl of solution containing ∼12.5 ng/µl of gRNA and ∼300 ng/µl of Cas9 mRNA. The next day, injected embryos were inspected under a stereoscope and were classified as dead, deformed or normal phenotypes. Only embryos that developed normally were assayed for target site mutations using T7 Endonuclease I assay or DNA sequencing (see below). Genomic DNA was extracted from either single embryos or from a pool of ten embryos, as previously described.

### Statistical analysis

Data are presented as mean±s.d. Statistical analyses were performed with either an analysis of variance (ANOVA) or an unpaired two tailed Student's *t*-test. All statistical analyses were performed using SPSS 16.0 software (IBM) and values of *P*<0.05 were considered statistically significant.

## Supplementary Material

Supplementary information
