# Supplementary material for: Prednisolone induces osteoporosis-like phenotypes via focal adhesion signaling pathway in zebrafish larvae
Source: Biol Open. 2018 Jul 15;7(7):bio029405. doi: 10.1242/bio.029405 (PMC6078353; doi:10.1242/bio.029405)

Table S1 statistical analyses of candidate reference genes and stability value.

Gene	CT-value	M-value
β-actin	20±0.6	0.28
ef1□	22±1.2	0.781
gapdh	18±2.3	1.327

Table S2

Gene name	Forward primer	Reverse primer	Note
itga10	TTGAAGCCGCCAAGAACATC	CTTTAGTGGCCCCTTCCCTC	qRT-PCR
itgbl1	TGCAACTGTGGCAAGTGTTT	AGCAAACTACACCCTGAGCA	qRT-PCR
itga10	GTTGGTGTCTTGCAGTATG	CAGGCCATTTGGATGGCGTA	Cas9
itgbl1	GCAGGTGGGTGGAGAGTGTAAG	CCCCACAGGTCTTTCCATCA	Cas9

Table S3

Click here to download Table S3

Table S4

Click here to download Table S4

Table S5

Click here to download Table S5

Biology Open • Supplementary information

Supplementary Fig. 1 The top20 KEGG signaling pathway enriched by difference expression genes.

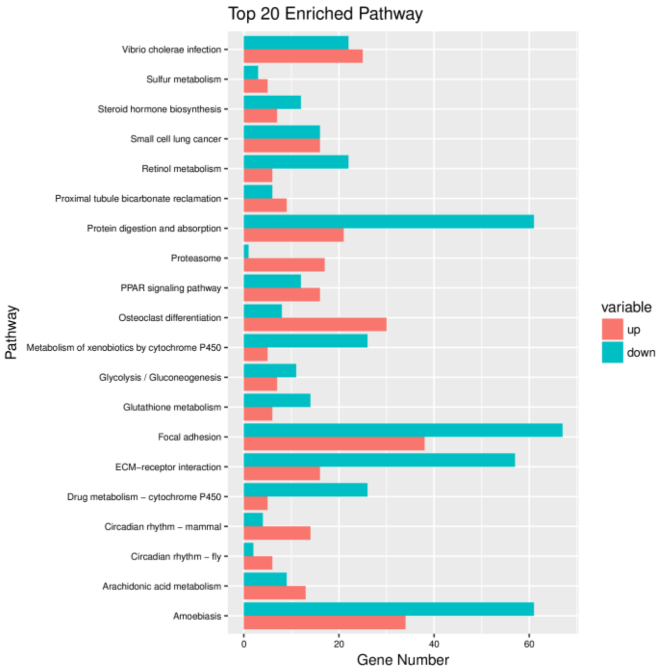

Biology Open • Supplementary information

Supplementary Fig. 2 The NF-KAPPA B signaling pathway enriched by difference expression genes.

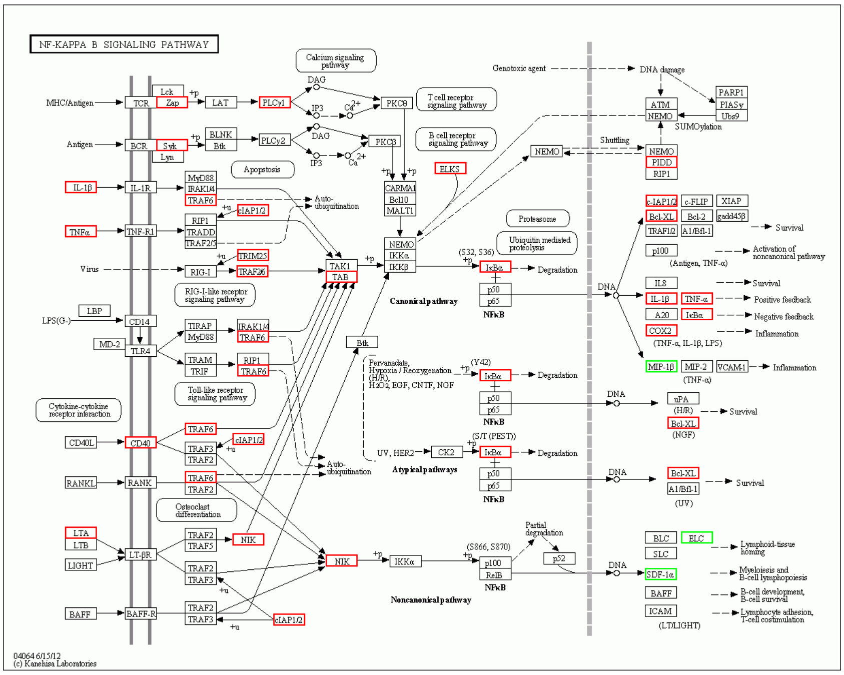

Supplementary Fig. 3 The osteoclast differentiation signaling pathway enriched by difference expression genes.

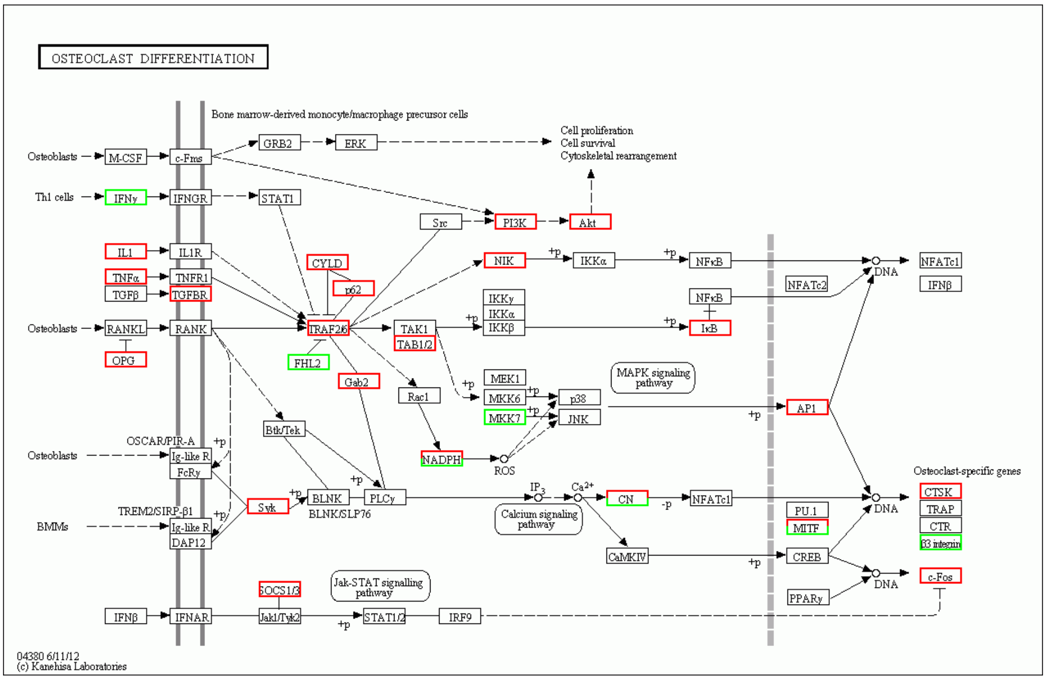

Biology Open • Supplementary information

Supplementary Fig. 4 The Wnt signaling pathway enriched by difference expression genes.

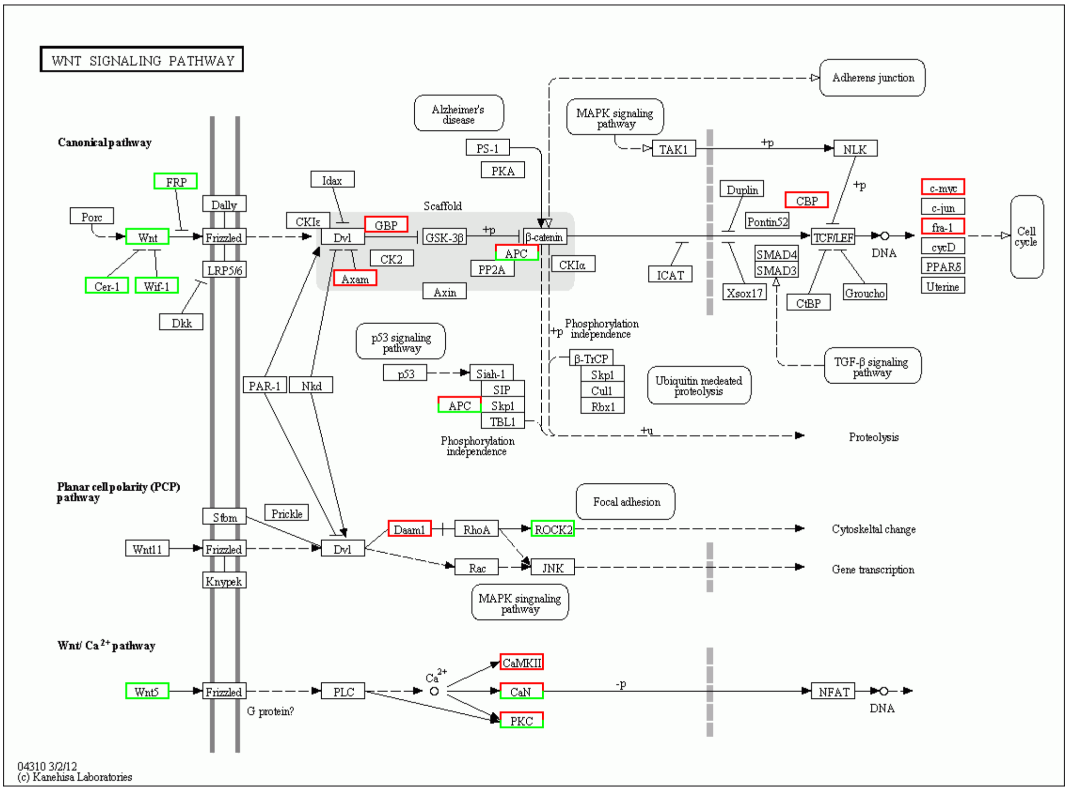

Supplementary Fig 5 the MAPK signaling pathway enriched by difference expression genes.

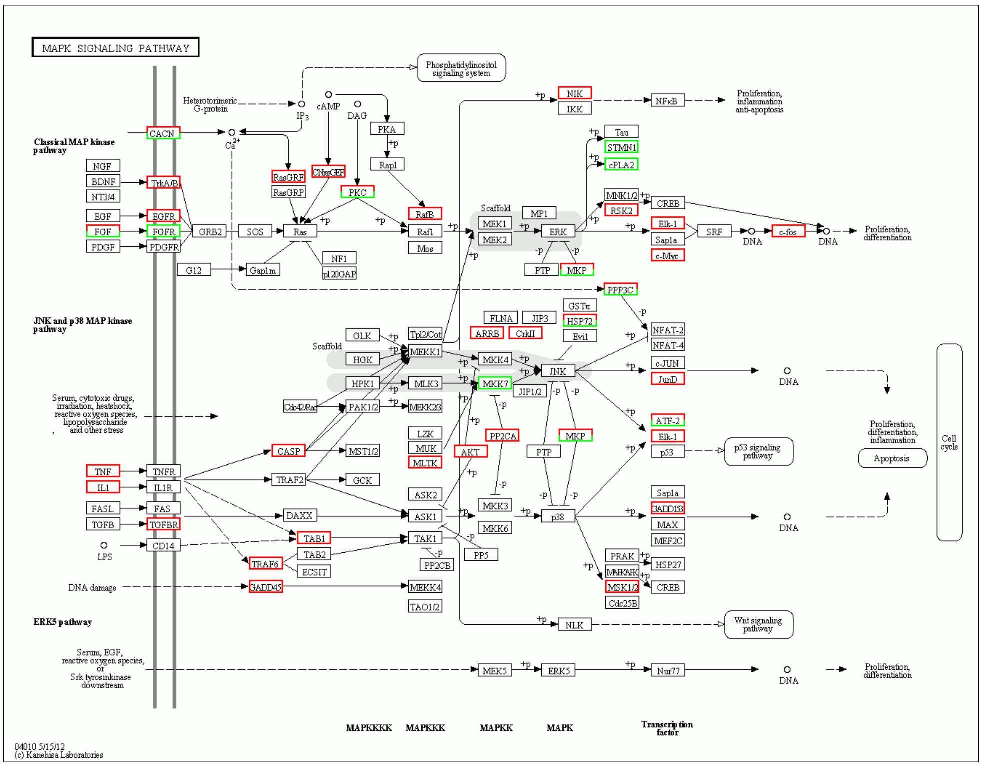

Biology Open • Supplementary information

Supplementary Fig. 6

Knockdown of *itga10* and *itgbl1* by *Cas*9. (A) The gRNA for the locus of *itga10* was targeted in exon 8 and efficiency was evaluated by Bsl1 digestion. (B) The gRNA for the locus of *itgbl1* was targeted in exons 2 and efficiency was identified by Bsl1 digestion.

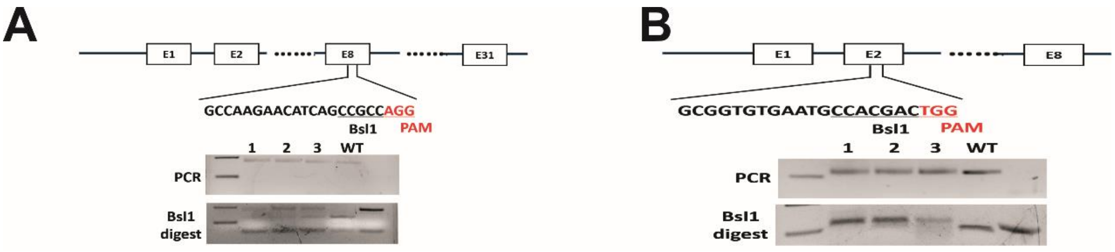

Supplementary Fig. 7

Capped mRNA was partly worked in the 10dpf larva fish. The *itga10* and *itgbl1* fused mCherry was injected eggs. We can founded weak but obvious mCherry fluorescence in the injected 10dpf fish.

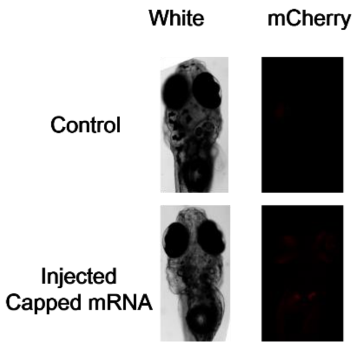

Supplement: Supplementary information [file biolopen-7-029405-s1.pdf]
